# An Overview of Bioinspired and Biomimetic Self-Repairing Materials

**DOI:** 10.3390/biomimetics4010026

**Published:** 2019-03-20

**Authors:** Olga Speck, Thomas Speck

**Affiliations:** 1 Plant Biomechanics Group and Botanic Garden, University of Freiburg, Schänzlestr. 1, 79104 Freiburg, Germany; thomas.speck@biologie.uni-freiburg.de; 2 Cluster of Excellence *liv*MatS @ FIT—Freiburg Center for Interactive Materials and Bioinspired Technologies, University of Freiburg, Georges-Köhler-Allee 105, D-79110 Freiburg, Germany

**Keywords:** self-sealing, self-healing, plants, animals, functional principle, damage control, damage prevention, damage management

## Abstract

During the 3.8 billion years of biological evolution, a multitude of functional principles has been developed in all kingdoms of life enabling the sealing and healing of diverse types of damage. Inspired by this treasure trove, biologists and engineers have become increasingly interested in learning from biological insights for the development of self-repairing materials. In this review, particular attention is paid to the systematic transfer of knowledge from wound reactions in biological role models to technical applications with self-repair function. This knowledge transfer includes bioinspiration in terms of the conscious implementation of an idea from nature or biomimetics in the form of a systematic transfer of underlying functional principles found in selected biological role models. The current overview presents a selection of breakthroughs regarding bioinspired or biomimetic self-repairing materials, including the initial basic publications and the recent publications of the last eight years. Each reviewed publication is presented with reference to three key criteria: (i) self-repair mechanisms in plants or animals as role models; (ii) knowledge transfer from living nature to technology; and (iii) bioinspired or biomimetic materials with self-repair function. Finally, damage control is discussed with a focus on damage prevention and damage management.

## 1. Introduction

The term “self-repair” seems to be a contradiction in itself. Composed of the words “self” and “repair”, it expresses the unimaginable, namely that the repair of damage is not carried out by a craftsman with the help of appropriate tools but takes place autonomously with the help of available means. Interestingly, in both biological and technical systems, the generic term “self-repair” can be further subdivided into an initial rapid phase of self-sealing and a subsequent slower phase of self-healing of wounds and damage [1,2]. In this review, the terms self-repair (overarching term), self-sealing and self-healing (two repair phases) are consistently used to describe the biological examples (Table 1). If the sealing phase of synthetic self-repairing materials is not considered separately, then they are simply referred to as self-healing materials. For this reason, the description of artificial materials cannot follow the nomenclature as consistently as in the case of biological models, especially when it comes to established fixed terms.

Wound reactions can be seen as a fundamental function of living nature. During the 3.8 billion years of biological evolution, various self-repair mechanisms have evolved independently and several times in plants, animals and all other groups of organisms [3]. However, self-repairing materials are not a privilege of nature. Even if the inspiration for self-repair is linked to nature in different ways, most existing self-repairing materials have been developed by engineers, chemists or material scientists without a deliberately chosen biological role model. Nevertheless, the transfer of biological insights to technical applications is possible because of the common physical, chemical and mathematical laws in biology and technology.

Among the scientists of various disciplines, a discussion has arisen about the way that self-repairing materials should be defined and characterized. Is self-repair a material property or a function? What test method can be used to analyze the self-repair effect quantitatively? What equation(s) should be used to calculate the efficiency of self-repair? Which standards should be agreed upon with regard to the type of injury (small to large damage), the duration of the repair process (seconds to days), the number of repetitions (once, often, unlimited), the type of repair process (autonomic or supported by external factors such as temperature, light, humidity, mechanical compression), the choice of the characteristic parameter examined (stiffness, strength, mass flow) and other boundary conditions such as geometric data (curvature, shape) [4,5]? In the following, these questions will be answered within the subject of the review. 

In the literature, one can find numerous definitions and classifications of self-repairing materials that are based on diverse criteria. One classification is made according to the basic types of processes involved resulting in the differentiation between chemical and physical self-repairing processes [6]. Another classification distinguishes between intrinsic self-healing, whereby healing takes place because of an inherent capability of the material and extrinsic self-healing, whereby healing chemicals are embedded in microcapsules, hollow glass fibers or vascular systems and delivered upon damage [6,7,8,9]. A third classification is made depending on the required trigger characterizing the nature of the self-healing process and thus distinguishes between non-autonomic self-healing materials, which necessitate a modest external trigger and autonomic self-healing materials, for which the damage itself is the stimulus for the healing [9]. The list can be continued at will but, with regard to the topic of this review, it should only be supplemented by the criterion of the existence of a biological role model as a basis for the development of self-sealing and self-healing materials. Various scenarios with the involvement of biological models can be distinguished. If living systems or the subsystems thereof are used directly, it is always a biotechnological product [10]. In the context of this review, self-repairing concrete is a good example of a biotechnological product, because the living bacteria contained in the concrete produce the metabolic product calcium carbonate and can thus seal cracks [11,12]. Biotechnological products stand in contrast by the direct involvement of living (sub)systems to bioinspired and biomimetic products in which living organisms are exclusively used indirectly in terms of an idea provider or a concept generator, respectively [11]. Irrespective of from which source the basis for a self-repairing material originates, one cannot evaluate its quality. Decisive quality factors include both the fulfilment of the self-repair function within the framework of the respective material requirement profile and its market readiness.

Currently, the literature contains various measurement methods and equations for evaluating the quality of self-repair. However, a comparison can only be made if exactly the same test protocol has been used both within material classes and between different material classes such as ceramics, concretes, brittle and elastic polymers, fiber-reinforced composites and biological materials. This is widely used to determine the percentage recovery of a selected mechanical property as a measure of the quality of the repair function. Mechanical tests (e.g., bending, tension, compression, torsion, cycling) determine the material properties of test specimens (e.g., stiffness, strength) before and after self-repair and are then used to calculate a dimensionless value expressing the percentage recovery of the respective mechanical property [6,7,9]. In most cases, the healing efficiency (η) of the mechanical properties is calculated as percentage of the property of the pristine material by using either Equations (1) or (2) [7,13,14]:

(1) $$\eta \;\left(\%\right)=100\;\left[\frac{healed\;property}{pristine\;property}\right]$$

(2) $$\eta \;\left(\%\right)=100\;\left[\frac{healed\;property-damaged\;property}{pristine\;property-damaged\;property}\right]$$

However, even if a single mechanical property regenerates to 100%, this does not make any statements about other mechanical properties [7], structural characteristics or even the functionality of the overall structure or, in other words, the quality or state of being functional [15]. However, for both injured biological and damaged artificial materials, we can assume that it is the functionality rather than the exact external or internal microstructure that has to be repaired [9]. One consideration is also of great importance in medicine, where the term *restitutio ad integrum* is used to describe complete functional and morphological restoration to the state before an illness or injury, whereas the term *reparatio* is used when a persistent defect remains after healing. Superficial abrasions of the skin undergo *restitutio ad integrum*, whereas any deeper injury leads to *reparatio*. The latter is characterized by the possible lack of glands, hairs, pigment cells and innervation and results in scaring and color changes in the former wound region [16,17,18]. Although the terms in the field of healing are defined differently in the individual disciplines, the self-repair function obviously depends on a variety of mechanical properties and structural characteristics that interact at various length scales and hierarchical levels, finally leading to complete or partial healing. Based on the natural model of self-repair in plants and animals as reference systems, it is all the more important to ensure comparability within and between different material classes by means of standardized measuring methods. In recent years, efforts have been made to assess self-healing efficiency when considering the specific characteristics of each material class and focusing on macro-, micro- and nanoscale damage events [12,14]. Furthermore, measurement standards should also consider whether the healing process can take place once, often or unlimitedly.

In this review, we present the state-of-the-art regarding bioinspired and biomimetic self-repairing materials. The aim of Section 2 is to demonstrate the variety of self-repair occurring in living nature. Both plants and animals represent complex materials systems in which self-sealing and self-healing can occur individually at each hierarchical level or can be the result of overarching mechanisms at various hierarchical levels. In this section, general self-repair principles are revealed based on various bauplans of plants and several bodyplans of animals. The scope of Section 3 is to describe the knowledge transfer from biology to technology. Based on a nature-inspired idea, the conscious transfer into a technical application leads to a bioinspired application. If functional principles are additionally transferred, then a biomimetic product is developed. The biologically inspired design (BID) and the biomimetic approach are characterized by a systematic step-by-step procedure. Section 4 covers characteristic examples of bioinspired and biomimetic self-repairing materials and takes into consideration both the initial basic publications and the recent publications of the last eight years. The focus of this section is the provision of detailed information concerning the respective biological role model (plants, animals, living organisms in general), the type of knowledge transfer (idea and/or functional principle), the developed product and its field of application. To conclude, the concept of damage control is discussed either as damage prevention or as damage management with respect to biological, bioinspired and biomimetic self-repairing mechanisms.

## 2. Self-Repair in Living Nature

**Figure 1:**
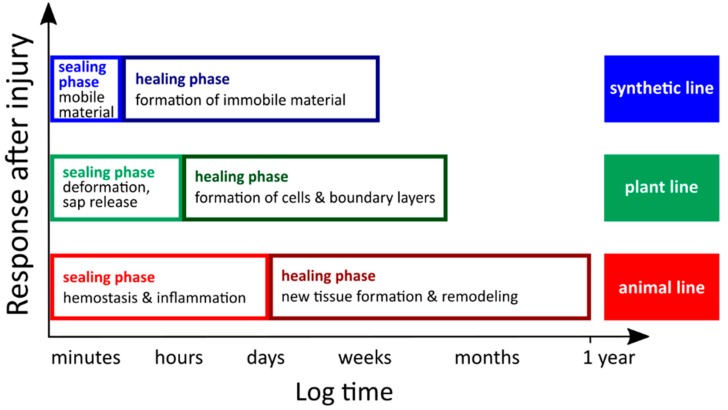
Comparative depiction of self-repairing phases in plants, animals and technical materials. Although the phases may vary in duration and the underlying mechanisms are different, they all have in common that the initial sealing phase guarantees wound or damage closure and the healing phase leads to a (partial) restoration to the uninjured state. Inspired by Blaiszik et al. [25].

In this overview, we restrict the self-repair of living nature to the self-sealing and self-healing mechanisms found in multicellular organisms. Although regeneration in terms of the regrowth of entire organs offers fascinating insights into nature, the exact means of activating regeneration in plants and animals are not detailed in this context, since the functional principles of the dedifferentiation of cells or the “freezing” of a state of developmental youth [19] are not suitable as role models for artificial self-repairing materials. This aspect is different from the self-repair mechanisms that occur in many higher plants and animals and that have recently proved to serve as suitable idea providers and concept generators. In general, self-repair mechanisms in higher animals and plants can be subdivided into an initial self-sealing phase and a subsequent self-healing phase (Table 1). Rapid self-sealing leads to a remaining injury (i.e., fissure, rupture, cut, crack) that is repaired functionally to such an extent that it can fulfil the previous function (e.g., tightness, self-cleaning), although the mechanical properties (e.g., stiffness, strength) are not completely restored. In contrast, the injury is structurally repaired after a long-lasting self-healing phase and is therefore no longer present. Additionally, the mechanical properties of healed materials are (at least partially) restored. Interestingly, this subdivision also holds true for both natural and man-made self-repairing materials systems [1,2,3,20]. With regard to biological models, plants and animals have been established as representing materials systems that are built up hierarchically. Thus, initial self-sealing and subsequent self-healing can take place individually at each hierarchical level or can be the result of overarching mechanisms at different levels, whereby the respective level components may be functionally redundant at other levels, may support one another or may combine supposedly conflicting functions [20,21]. The potential for efficient self-repair also depends on the contrasting specific system properties in plants, which are characterized by continuous growth and those in animals whose development is completed with sexual maturity [22,23]. Among others, the multitude of hierarchical levels with their diverse structures and components and the differences in their system characteristics have revealed that plants and animals have developed different self-repair mechanisms during biological evolution. Remarkably, a common pattern of self-repair can be observed in higher plants and animals [19,24] and in artificial self-repairing materials. Even if the time spans and mechanisms are different, all systems involve an initial self-sealing phase that leads to rapid wound or damage closure distinguishable from a later self-healing phase that leads to the most precise possible restoration of the initial state (Figure 1).

### 2.1. Self-Repair in Plants

Vascular plants (tracheophytes), which are typically sessile and therefore cannot escape from unfavorable environmental conditions, have evolved a variety of morphological, anatomical and biochemical adaptations to survive (sometimes severe) disturbances. These evolutionary traits include self-repair and defense mechanisms as reactions to injuries of various kinds. Pure mechanical damages may occur by the trampling or feeding animals, rock falls, falling plant organs or other air-borne parts and overcritical bending or twisting in strong storms [26]. A combination of mechanical damage and chemical attack can be assumed after insect herbivory. In the latter case, plants respond by direct defense, namely affecting the physiology and/or behavior of the attacking insects and by indirect defense, such as the attraction of natural enemies of the herbivores [27,28]. The current review will focus on mechanically driven wound reactions of plants characterized by their specific bauplans. In connection with self-repair in vascular plants, the following three aspects are of particular interest. First, plants are described as open systems, characterized by the permanent presence of “embryonic” stem cells in the growth zones (e.g., vegetation points, cambia). This enables plants continuously to grow and form new organs until death [22,23]. Second, in vascular plants, at least five hierarchical levels can be found from the macroscale to the nanoscale: (i) organs (e.g., roots, leaves, stems); (ii) tissues (dermal, ground and vascular tissue); (iii) cells (e.g., parenchyma cells, vessels, fibers, laticifers); (iv) organelles (e.g., nucleus, chloroplasts, mitochondria); and (v) molecules (e.g., cellulose, hemicellulose, pectin, lignin, latex) [20,21]. Hierarchical structuring offers the potential to achieve self-repair at each single level or by the combination of several levels whereby every single level can promote the self-repair function or even solve conflicting functional requirements [29]. Third, during self-repair processes in all the plant species investigated so far, an initial rapid self-sealing phase and subsequent long-term self-healing phase can be discerned (Figure 1) [1,2,3].

#### 2.1.1. Self-Sealing and Self-Healing Mechanisms of Plants

**Figure 2:**
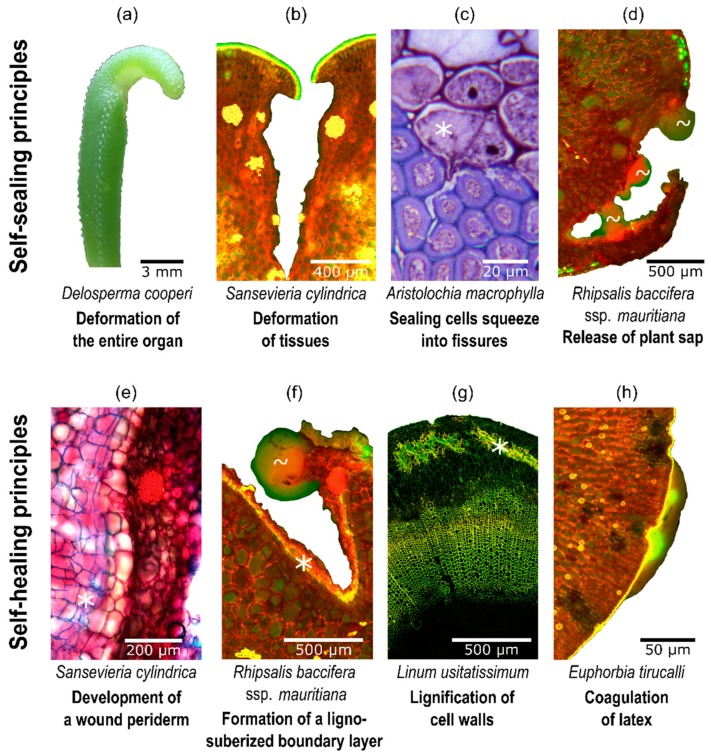
Examples of plant species showing various types of wound reactions. (**a**) The bending of the entire leaf sealing the wound within approximately 60 min after the damage and subsequent wound healing over a longer time period (days or weeks). (**b**) The rolling-in of the dermal tissues at the wound edges (epidermis with cuticle) reduces the size of the wound opening. (**c**) Thin-walled parenchyma cells (*) deform and squeeze into the (micro)fissures of the thick-walled and lignified sclerenchyma cells. (**d**) Injuries are immediately sealed by mucilage (~) released from destroyed mucilage cells in the wound region. (**e**) Development of a wound periderm including cell division (*). (**f**) Formation of a ligno-suberized boundary layer around the wound (*), mucilage discharge (~) covering the wound surfaces having taken place in the sealing phase. (**g**) Lignification of formerly nonlignified cell walls of bast fibers (*) in the wound area. (**h**) Release of latex immediately after the injury, which forms a permanent plug of coagulated latex within minutes. (**b**,**d**,**f**,**g**,**h**) Thin sections stained with acridine orange, which highlights lignified structures in bright yellow-green; (**c**) cross-section stained with toluidine blue, which highlights lignified structures in blue; (**e**) thin-section stained with fuchsin-chrysoidine-astra blue according to Etzold after which lignified cell walls appear bright red and nonlignified cell walls are highlighted in blue.

Figure 2 shows some selected examples of self-repair mechanisms in vascular plants. Self-sealing is mainly based on physical reactions and leads to a functional repair of the fissures that are still present (e.g., superficial wound closure, covering of inner and outer wound surfaces). Self-sealing can occur within a few minutes via the discharge of plant saps (e.g., latex, resin, mucilage) that fill and seal the gap and/or within minutes to hours via mechanically driven deformation bringing together the wound edges (e.g., overlapping of wound sites, close contact of wound surfaces, rolling-in of wound edges). Self-healing is mainly based on chemical reactions and biological responses leading to a structural repair of the fissures that are then no longer present (regaining mechanical properties, defense mechanism). Self-healing is in general related to the formation of a (ligno-) suberized boundary layer, to the development of a wound periderm that induces cell division lasting from several days to weeks and in the case of latex-bearing plants, to the coagulation of latex within minutes [3,26,28].

#### 2.1.2. Wound Responses of Plants at Various Hierarchical Levels

Table 2 gives an overview of the self-sealing and self-healing mechanisms of vascular plants arranged according to the various hierarchy levels. Since plants and plant organs can be considered as fiber-reinforced composite materials that consist of lignified dead fibers (sclerenchyma) and/or nonlignified living fibers (collenchyma) embedded in lignified and/or nonlignified matrices (parenchymatous tissue), the three-dimensional arrangement of the various cells and tissues is of utmost importance for the self-repairing efficiency. The described anatomical heterogeneity causes the mechanical anisotropy of the plant organs. Therefore, the wound reaction is different depending on the orientation of the wound [30]. Among other features, the bauplans of vascular plants differ considerably with respect to the arrangement of fibers and vascular bundles in the stem. In stems of herbaceous monocotyledonous plants, numerous vascular bundles are scattered in the ground tissue and undifferentiated cambium cells for additional cell formation are rarely present. In contrast, stems of dicotyledonous plants possess a ring of vascular bundles or even a closed circular ring of xylem and phloem with a vascular cambium in-between (a cortex cambium additionally exists in woody species).

### 2.2. Self-Repair in Animals and Humans

Unlike sessile plants, most animals have evolved a fascinating range of adaptations that allow them to be motile by walking, swimming or flying. On the one hand, this enables them to avoid dangerous situations and possible injuries. On the other hand, this also challenges them with new dangers for injury, such as collisions with dead or living objects, turf warfare or bite wounds. Interestingly, two macromolecules of the cytoskeleton (microtubules and microfilaments) ultimately generate the cell movement that also plays a crucial role in cutaneous wound repair during wound contraction [31]. Some similarities and dissimilarities can be observed when comparing wound reactions of vertebrates (tetrapodes) and vascular plants (tracheophytes) [19,24]. Three aspects are of particular interest in the wound response of vertebrates. First, vertebrates are, in general, described as closed systems because the growth of higher animals is severely restricted to a growing phase or completed at sexual maturity [22]. Under these conditions, it is all the more astonishing that some animals can regenerate entire extremities. An interesting example is the repeatable limb regrowth of salamanders (bodyplan of amphibian) [3]. Depending on the respective bodyplan of animals, regeneration of entire organisms (as also found in plants) can even serve as a means of asexual reproduction (e.g., sponges as members of the Porifera, planarians as members of the Platyhelminthes and asteroids belonging to the Echinodermata) [24,32]. Second, vertebrate organisms are hierarchically structured as are those of higher plants. However, vertebrates possess at least six hierarchical levels: (i) organ systems (e.g., respiratory system, digestive system, skeletal system); (ii) organs (e.g., skin, bone); (iii) tissues (e.g., muscle tissue, epithelial tissue); (iv) cells (e.g., muscle cells, osteoblasts, osteoclasts); (v) organelles (e.g., nucleus, mitochondria); and (vi) molecules (e.g., actin, myosin, microtubules, collagen) [20,21]. Third, the wound response of vertebrates also shows a rapid self-sealing phase (so-called early phase) and a subsequent self-healing phase (so-called cellular phase) (Figure 1) [33]. Despite these similarities, wound repair in various phyla show tremendous diversity [33]. However, in the context of the research relevant for this review, human wound healing of skin or bone have been used as the primary role models used for the development of animal-inspired self-healing materials. Therefore, only these examples are presented in more detail in the following section. Interestingly, in all organ systems, mammalian wound response can be divided into three overlapping but distinct stages: (i) hemostasis and inflammation; (ii) proliferation and maturation; and (iii) remodeling.

#### 2.2.1. Human Wound Reactions to Skin Injury

**Figure 3:**
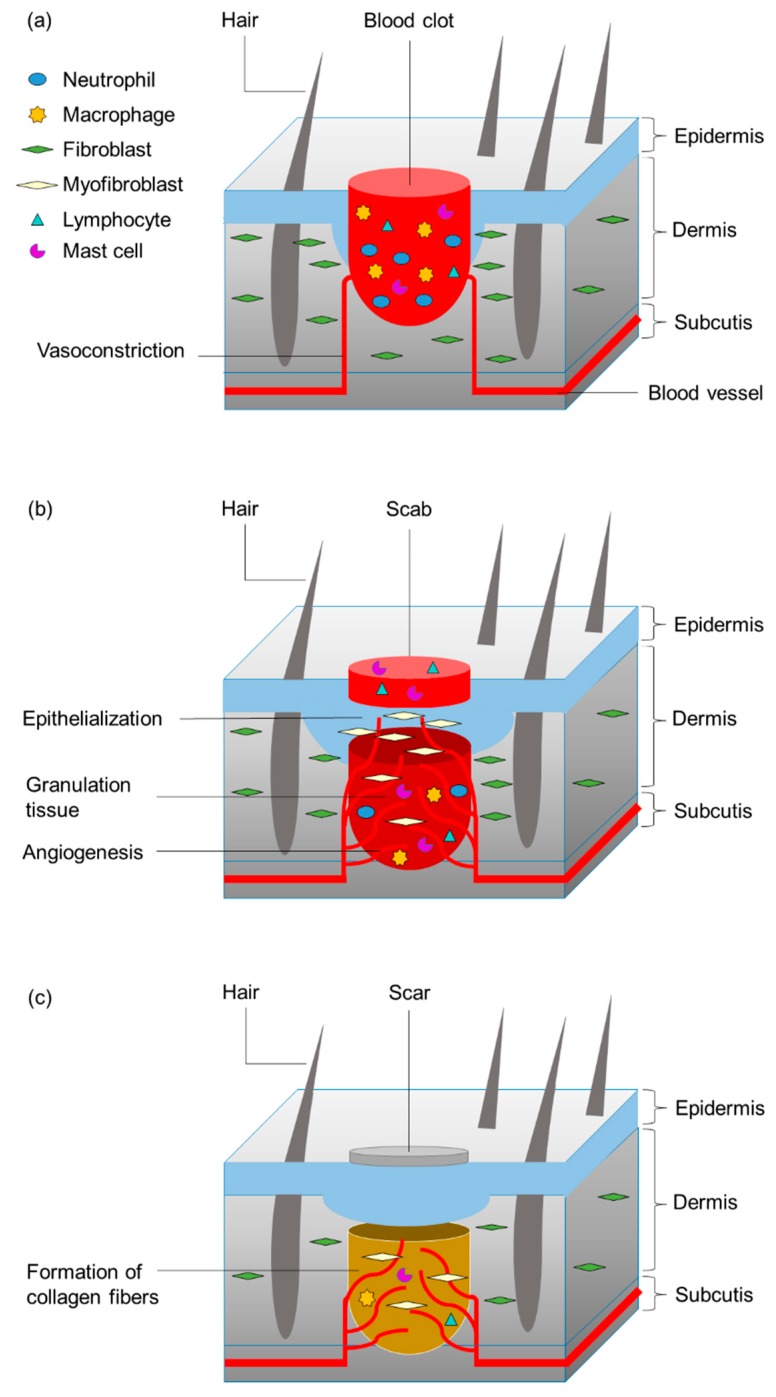
Phases of human wound reaction after a full-thickness skin injury. (**a**) Hemostasis and inflammation; (**b**) proliferation and maturation (new tissue formation); and (**c**) remodeling (with scarring).

Human wound reaction after a full-thickness skin wounding first shows a hemostasis and inflammation phase that begins with the injury and lasts up to 2–5 day after the injury, followed by a proliferation and maturation phase that lasts for 5 days to 2 weeks after injury and then enters the phase of remodeling (1–12 months after injury) (Figure 3). Immediately after an injury, vascular endothelial cells vasoconstrict to prevent further blood loss. The wounds are sealed by blood clotting. The clot attracts inflammatory (e.g., neutrophils) and repair cells and releases cytokines that initiate the inflammatory response. Macrophages clear the wound of pathogens and dead cells to prevent infection. Additionally, fibroblasts and endothelial cells present on the margins of the wound are activated. The subsequent proliferation stage starts with the formation of granulation tissue bridging the gap between the wound margins. In the maturation stage (epithelialization), the deposition of temporary extracellular matrix and the formation of blood vessels (angiogenesis) occur. Moreover, some fibroblasts differentiate into myofibroblasts, which are contractile cells that approach the wound edges. Fibroblasts and myofibroblasts produce an extracellular matrix, composed mainly of collagen, which forms the bulk of the scar. The healing phase is completed with the remodeling stage, during which most cells undergo programmed cell death (apoptosis). This preserves the extracellular matrix consisting of a few remaining cells but mostly of collagen and other extracellular proteins. The immature collagen fibers are replaced by mature collagen fibers. The consequent cross-linking of the mature collagen fibers results in a strengthening of the repaired tissue (e.g., scar formation). Nevertheless, the repaired tissue never regains the original properties and structure of intact skin, because of the changes in its fiber architecture [17,18,33,34].

#### 2.2.2. Human Wound Reactions to Bone Fracture

**Figure 4:**
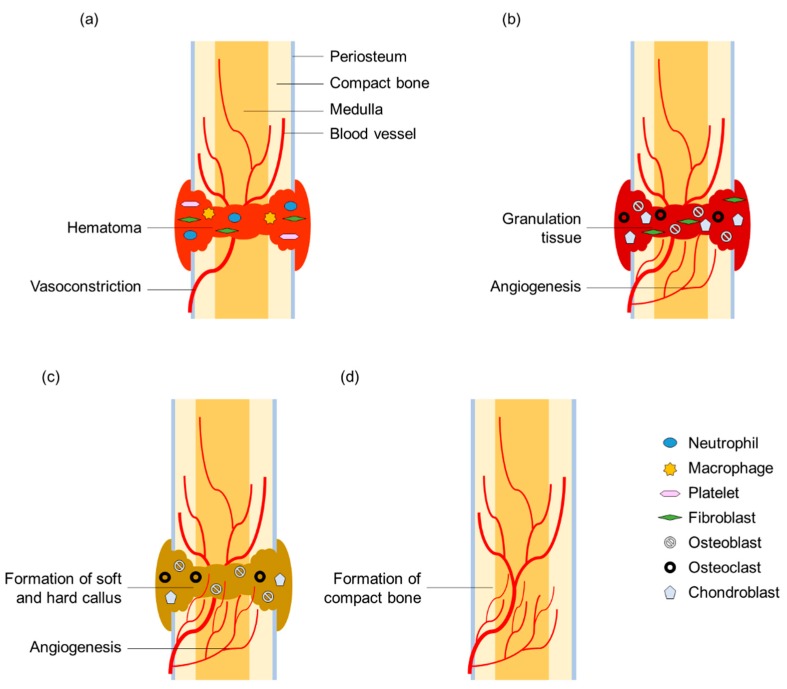
Phases of indirect (secondary) healing after bone fracture in humans. (**a**,**b**) Reaction involving (**a**) hemostasis, inflammation and (**b**) granulation tissue formation; (**c**) repair involving the formation of cartilage callus and lamellar bone deposition; and (**d**) remodeling (restoration of the original bone contour without scar formation).

After an initial trauma, fractured bone can heal either by direct intramembranous healing or indirect fracture healing. Here, only the most common pathway of indirect (secondary) bone healing will be described. In principle, the healing of bone fractures also displays three phases of wound reaction: (i) the reaction phase, including hemostasis and inflammation, begins with the fracture and peaks at 24 h after injury; (ii) a repair phase follows including callus formation, which lasts for 2 weeks after fracture; and finally (iii) the phase of bone remodeling takes place (up to 7 years after fracture) (Figure 4). Triggered by the trauma, an accumulation of blood cells in the region of the bone fracture and the constriction of blood vessel occurs collectively preventing further bleeding. Subsequently, a hematoma develops, which serves as a model for the later callus formation. During the inflammation stage, macrophages, neutrophils and platelets release numerous cytokines that mediate the recruitment of mesenchymal stem cells and their subsequent differentiation into osteoblasts and chondrocytes. Necrotic tissues are cleared by osteoclasts, whereas osteoblasts and fibroblasts proliferate. Within 7 to 14 days, granulation tissue, which is a loose aggregate of the aforementioned cells interspersed with small blood vessels (angiogenesis), forms around the fracture ends. The following callus formation, which peaks at day 14 after fracture, is divided into two parts. First, soft callus or hyaline cartilage is formed by chondroblasts. Second, hard callus or woven bone is formed by osteoblasts. Together, they represent the so-called fracture callus, a heterogeneous tissue that can bridge the gap between the fracture ends. This mineralized matrix fracture callus is then penetrated by microvessels and osteoblasts and the woven and hyaline bone is consecutively replaced by lamellar and trabecular bone (endochondral ossification). Throughout the year-long process of remodeling, the bone adapts to the mechanical stresses to which it is exposed. During this process, osteoclasts remove bone material and osteoblasts deposit compact bone within the resorption pit (known as Howship’s lacuna), thus remodeling the bone according to the prevailing lines of force. Interestingly, the fracture healing of bones shows no fibrous scar and can therefore be described as *restitutio ad integrum* [35,36]. 

#### 2.2.3. Human Wound Responses at Various Hierarchical Levels

Although in the literature, the names of the phases after an injury sometimes differ slightly, the dynamic process of wound response is divided into three phases and the same or comparable mechanisms of wound response are described, all of which can be found successively or simultaneously at various hierarchical levels. Table 3 gives an overview of self-sealing and self-healing mechanisms found in human wound reaction after skin injury and bone fracture. 

## 3. Bioinspired and Biomimetic Approaches

Even a simple observation in nature can lead to a targeted transfer of the inspiring idea and thus to the development of a bioinspired material with self-repair function. Based on further systematic investigations of the biological role model, the underlying functional principles of the self-repair function can be decoded and finally transferred to technical applications. Irrespective of the chosen approach, learning from biological solutions for technical applications always requires a wide interdisciplinary cooperation of scientists such as biologists, chemists, physicists, mathematicians, materials scientists, designers, architects and engineers. However, the basic prerequisites for the transfer of biological insights into technical developments are the common physical, chemical and mathematical laws existing in both biology and technology. Based on this common framework, a systematic and stepwise approach can be applied that differs less in the type of steps than in the type of knowledge transferred (e.g., the transfer of an idea, a functional principle, the morphology or the application of a biomimetic algorithm) [10]. The biologically inspired design [37,38] and the biomimetic approach [39,40,41] belong to the best-known and most used methods. 

### 3.1. Biologically Inspired Design

#### 3.1.1. Brief Description

The BID uses analogies to biological systems to develop solutions for design problems. In principle, a problem-driven process and a solution-driven process can be distinguished [37,38].

#### 3.1.2. History of Origin

In 2006, a descriptive account of BID was conducted in the context of an undergraduate classroom setting at Georgia Tech, Atlanta, GA, USA. An interdisciplinary team of 4–5 designers, with at least one designer having a biology background and a few from different engineering disciplines, was supported by lecturers with many years of experience with BID. The designer team identified a problem that could be addressed by a biologically inspired solution, explored a number of solution alternatives and finally designed one or more biologically inspired designs.

#### 3.1.3. Problem-Driven Biologically Inspired Design Process

The pattern of problem-driven BID follows a progression of steps starting with the technical problem to be solved. The individual steps comprise:

- Step 1: problem definition
- Step 2: reframing of the problem
- Step 3: search for biological solutions
- Step 4: definition of the biological solution
- Step 5: principle extraction
- Step 6: principle application [37,38]

Since this is a dynamic development process, feedback and refinement loops need to be provided. 

#### 3.1.4. Solution-Based Biologically Inspired Design Process

The pattern of solution-based BID also follows a sequence of steps but contrary to the problem-driven BID, the starting point here is a particular biological solution. The individual steps comprise:

- Step 1: identification of the biological solution
- Step 2: definition of the biological solution
- Step 3: principle extraction
- Step 4: reframing of the problem
- Step 5: problem search
- Step 6: problem definition
- Step 7: principle application [37,38]

Similar to the problem-driven BID process, this highly dynamic process is not necessarily ordered linearly.

### 3.2. Biomimetic Approach

#### 3.2.1. Brief Description

By means of the biomimetic process, functional principles found in biological models are transferred to technical applications. In principle, a bottom-up approach and a top-down approach can be distinguished [39,40,41,42,43].

#### 3.2.2. History of Origin

In 2006, biomimetics experts from various fields of expertise (basic and applied research, and industry) and professions (biologists, chemists, physicists, mathematicians, materials scientists, designers, architects, and engineers) were invited by the Association of German Engineers (Verein Deutscher Ingenieure (VDI)) to collaborate on a number of VDI guidelines on selected topics of biomimetics. Some selected VDI guidelines have meanwhile been further developed into International Organization for Standardization (ISO) guidelines by international scientists. In the thematic context of this review, the guidelines concerning the concepts and strategy of biomimetics [40,41] and on biomimetic materials, structures and components [42,43] are of particular interest.

#### 3.2.3. Top-Down Approach and Bottom-Up Approach

**Figure 5:**
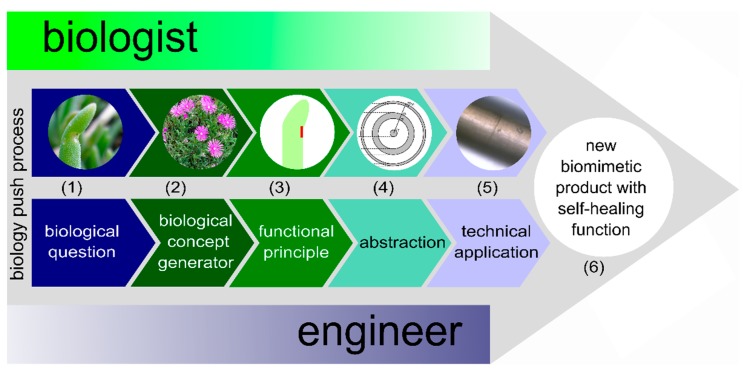
The bottom-up approach (biology push process) of a project aimed at revealing biological self-repairing functions. (1) What is the basis of the self-sealing function of an entire plant organ? (2) The Pink Carpet (*Delosperma cooperi*) has proved to be a suitable concept generator. (3) An effective self-sealing function by internal deformation has been found in the succulent leaves of *D. cooperi.* (4) The underlying sealing principle is a combination of hydraulic shrinking and swelling as the main driving forces and growth-induced mechanical prestresses in the five tissue layers also acting as a speed-boost mechanism. (5) Inspired by the mechanically driven deformation of the plant leaves, a polymer with a shape-memory effect was developed that finally leads to self-healing (reprinted from [45], Copyright 2018, with permission from Elsevier). (6) Since the leaf-inspired polymer has only recently been developed, a specific application is not yet available. Slightly modified and reproduced with permission from [4] Copyright © 2015.

**Figure 6:**
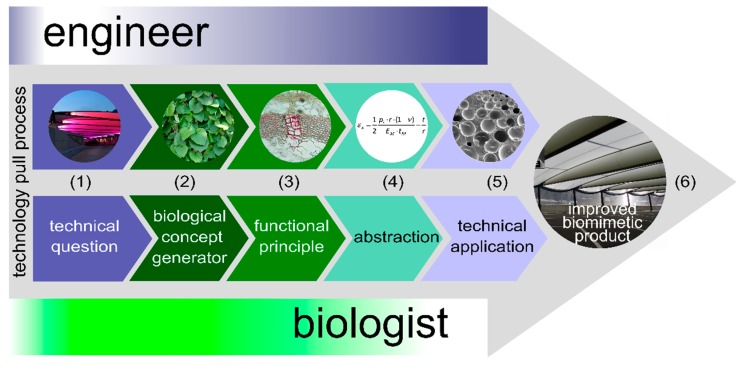
The top-down approach (technology pull process) of a self-sealing foam membrane coating for pneumatic structures. (1) How can fissures in the membranes of pneumatic systems such as the Tensairity^®^ technology (Airlight Ltd., Biasca, Switzerland) technology be rapidly closed? (2) The Dutchmen’s Pipe (*Aristolochia macrophylla*) has proved to be a suitable role model. (3) Growth-related (micro)fissures in the peripheral strengthening tissue of the stems are rapidly closed by sealing cells. The underlying functional principle is based on turgescent (under internal overpressure) nonlignified parenchyma cells that swell into the fissures. (4) Inspired by these turgescent sealing cells, a polyurethane foam coating was applied to the membrane on the inside of the pneumatic structure. The self-sealing foam consists of closed-cells and is polymerized under an overpressure of 1–2 bar. The interaction of the material properties and geometric parameters of the coated membrane can be described by a mathematical equation. (5) The self-sealing foam was tested and further developed in cooperation with an industrial partner on a pilot plant scale. (6) Membranes equipped with the commercially available foam can be incorporated into a multitude of pneumatic systems. Slightly modified and reproduced with permission from [4] Copyright © 2015.

The top-down approach is a stepwise development starting with a question from the engineering sciences for solving a technical challenge. Therefore, the top-down approach is sometimes also called the technology pull process. The bottom-up approach is also a systematic stepwise developmental process but with the difference that it starts with a question from biology for understanding biological systems. For this reason, the bottom-up approach is also known as the biology push process. As the two biomimetic approaches differ essentially in the first step, whereas the following steps differ only in a few details, they are presented together in Table 4 in which the individual steps and a brief description of each single step are given. Even if the biomimetic approaches are represented as linear processes in Table 4, and in Figure 5 and Figure 6; biomimetics is a highly dynamic and interdisciplinary heuristic spiral that can include several feedback loops. In addition, within each biomimetic approach, not only is a technical application created but also an in-depth understanding of the form–structure–function relationship of the selected biological models occurs, a knowledge gain that is coined as *reverse biomimetics* [39]. At this point, the *going well beyond biology* concept should also be addressed [2]. On the one hand, the biomimetic solution can be combined from several role models, even from the plant and animal kingdoms, which do not occur together in nature. On the other hand, the biomimetic technical system can be used in environments where biological systems would clearly fail and/or further functionalities that do not exist in living nature may be added to fulfil the desired final function of the technical application.

The great potential of a bottom-up project can be seen because new functional principles, which are largely unknown in technology up to then or have not been used for a certain task, are often recognized and thus represent a high level of inventiveness or scientific progress. A well-known example is the development of products with the Lotus-Effect^®^ (Sto SE & Co. KGaA, Stühlingen, Germany), inspired by the self-cleaning function of lotus leaves (*Nelumbo nucifera*), this example introduced a paradigm shift. An example with reference to the subject of the present review is the development of artificial materials inspired by the self-sealing mechanism found in the succulent leaves of *Delosperma cooperi* [44]. Figure 5 illustrates the bottom-up approach and introduces the individual steps that have been carried out. The findings of these studies have recently been applied with regard to the development and production of a self-healing polymer [45]. More details are given in Section 4.1.1 [44,45,46,47].

The top-down approach is defined as a “biomimetic development process in which an existing functional technical product is provided with new or improved functions through the transfer and application of biological principles” [40,41]. Since a specific application-based question from industry or engineering sciences rather than a basic research-oriented question initiates the project, the inventive level and scientific progress are usually lower than in bottom-up projects. The advantage of a top-down project lies in its usually relatively short development time of typically 2 to 4 years, which fits well within the planning periods that are customary in the industry. Figure 6 represents a plant-inspired foam coating for membranes used in pneumatic systems. Detailed information about the development of the self-sealing foam by scientists in a research and development project with the participation of an industrial partner is given in Section 4.1.4 [48,49,50,51,52,53].

## 4. Self-Repairing Materials Inspired by Nature

In living nature, self-repair occurs as a matter of course. Therefore, the observation of nature is assumed to have always inspired scientists and engineers in the development of self-repairing technical materials and materials systems. The recent increased interest in self-repairing materials and the associated systematic approach to implementing this function in a wide variety of materials can be seen, among other things, by the increase in scientific articles on this topic. From 2006 to 2014, the total number of papers of scientific publications on self-healing materials increased from 73 to 3954 [14]. As early as 1969, the self-healing of cracks was investigated and published, for the first time, in Mekhanika Polimerov by Malinskii et al. [54]. Some publications followed on the self-healing function in various material classes until, finally, the article of White et al. [55] appearing in *Nature* in 2001 attracted great attention to self-healing materials. Here, the authors emphasized, “We expect that the field of self-healing, although still in its infancy, will evolve beyond the method presented here until true biomimetic healing is achieved by incorporating a circulatory system that continuously transports the necessary chemicals and building blocks of healing to the site of damage.” [55]. In 2005, Pang and Bond [56] presented such a biomimetic tracking system with a self-repairing function. After damage by four-point bending, so-called “bleeding composites” performed both the visual enhancement of the damage by the bleeding action of a fluorescent dye and a significant restoration of strength of approximately 97% of the initial values by a healing agent that was stored in hollow fibers and released upon damage (see Section 4.2.4).

To date, increasing numbers of scientists are working on the development of self-repairing materials. These scientists include not only engineers and chemists but also botanists and zoologists who contribute their knowledge obtained from living nature. Whether a material has been conventionally developed or is bioinspired/biomimetic cannot be seen from the material itself. This is especially true of simple bioinspiration, the biomimetic approach or BID, as no design language emerges that can, with certainty, provide information about the flow of inspiration from biological role models [57]. For this reason, all development stories must be supported by a literature research and/or interviews with the respective scientists. Table 5 gives a chronological overview of the most recent ones from 2018 back to the initial basic publications. Publications that assumed that the self-repair function was omnipresent in living nature and that therefore could not provide any concrete information concerning the knowledge transfer from the biological model to the technical material were not considered. The bioinspired and biomimetic self-repairing materials and materials systems presented in Section 4.1, Section 4.2 and Section 4.3 are a selection of characteristic examples in which the flow of inspiration from the biological role model to the technical product could be traced and presented in a balanced form. Pure analogies between biology and technology or a later reinterpretation (*a posteriori biomimetization*) of the technical product as bioinspired or biomimetic are not taken into account. 

### 4.1. Plants as Idea Providers or Concept Generators

#### 4.1.1. Self-Healing Polymer Inspired by *Delosperma cooperi* Leaves

During biological evolution plants have evolved various adaptations to arid environments including the storage of water in the parenchyma of succulent plant organs and the minimization of damage-prone water loss by rapid sealing and the subsequent healing of injuries [26]. As shown in Figure 5, the rapid self-sealing principle of *D. cooperi* is the deformation of the entire leaf until the wound edges meet. After a longitudinal or transversal cut, the leaf bends and in the case of a circumferential cut, a contraction occurs in the incision region. After approximately 60 min, the self-sealing movement is completed and self-healing begins, favored by the direct contact of the wound surfaces and wound edges. Since rapid and efficient wound sealing is essential for the water balance of plants growing in water-poor areas, this sealing process is secured several times by redundant mechanisms. Self-sealing in leaves of the Pink Carpet plant is a combination of hydraulic shrinking and swelling as the main driving forces and of growth-induced mechanical prestresses. Since the five tissue layers of the leaf are alternately pretensioned and precompressed, an injury causes a mechanical imbalance and the leaf deforms until a new mechanical equilibrium is attained [1,2,3,4,44,45,46,47].

**Figure 7:**
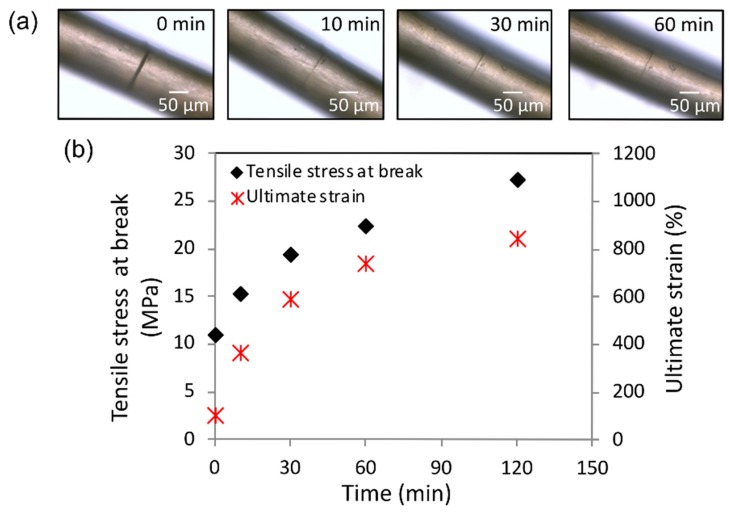
Self-healing of microphase-separated cold-drawing programmed polyurethane (PURP) fibers. (**a**) Optical images of PURP fiber. (**b**) Tensile stress at break and ultimate strain plotted as a function of healing time. Undamaged samples have an ultimate strain of 1015% and a tensile stress at break of 29.2 MPa. Values are the average of *N* = 6. Reprinted from [45], Copyright 2018, with permission from Elsevier.

Inspired by the above-described, mechanically driven, self-sealing principle in the succulent leaves of *D. cooperi*, Yang et al. [45] developed a bioinspired self-healing polymer. Based on the biological role model comprising of various tissues with inherent stresses and strains, they developed a commodity microphase-separated copolymer with consciously introduced morphologies facilitating shape memory effects (SME) that enable self-healing upon mechanical damage. The healable polycaprolactone–polyurethane polymer fibers drawn into fibers from solution during polymerization (cold-drawing programmed polyurethane fibers (PURP)). Figure 7 shows that, at a temperature of 65 °C, a scratch of 8 µm × 50 µm (width × depth) is optically healed within 10 min. Compared with undamaged samples, the mechanical properties such as tensile strain (35.8%) and stress at break (52.5%) are partly restored after 10 min and reach, on average, 82.6% and 87.0%, respectively, after 120 min. Comparative experiments with nanophase-separated copolymers (melt-drawn polyurethane fibers (PURM)) indicate a relationship between the degree of phase separation and shape recovery. The results of all experiments carried out clearly indicate that elastic energy stored in microphase-separated polymers (PURP) is released and that shape recovery takes place, which is ultimately responsible for damage closure [45].

In the framework of a bottom-up approach, the mechanically driven self-sealing principle found in leaves of the Pink Carpet has been transferred to a microphase-separated polymer with an in-built shape-memory effect being the basis for wound closure. Interestingly, here, only one principle of the redundantly secured sealing process, consisting of two principles, was successfully transferred from nature to an artificial material. Often, the opposite can also be found, namely that several biological models are combined in one technical product (see Section 4.1.3).

#### 4.1.2. Self-Sealing Wax System Inspired by *Banksia* Follicles

In harsh environments, such as fire-prone regions, plants have evolved special structures and mechanisms either to protect individual plants from fire damage or to ensure species conservation by adaptations of seed distribution. A meaningful example is the Australian plant genus *Banksia*, whose seeds are protected in woody fruits (follicles) consisting of two valves. After being exposed to heat or fire, the valves split along the suture and release the seeds. Huss and colleagues [59,60] investigated various species of *Banksia* and found that they incorporate waxes at the interface of the two valves of the follicle enclosing the seeds. In general, the waxes melt between 45 and 55 °C, temperatures that can easily be reached on a hot summer day in a natural location. The melting temperature is thus significantly lower than the opening temperature of the follicles of more than 68 °C. Therefore, they hypothesize that microfissures along the suture of the closed valves are sealed, a feature that contributes to the structural integrity of the fruit. 

In the framework of a bottom-up approach or solution-based BID, the functional principle of temperature-induced self-sealing, as found in the biological model *Banksia*, has been transferred to a simplified model system consisting of wood platelets sealed with melted carnauba wax.

#### 4.1.3. Self-Healing Coatings Inspired by *Lotus* Leaves

**Figure 8:**
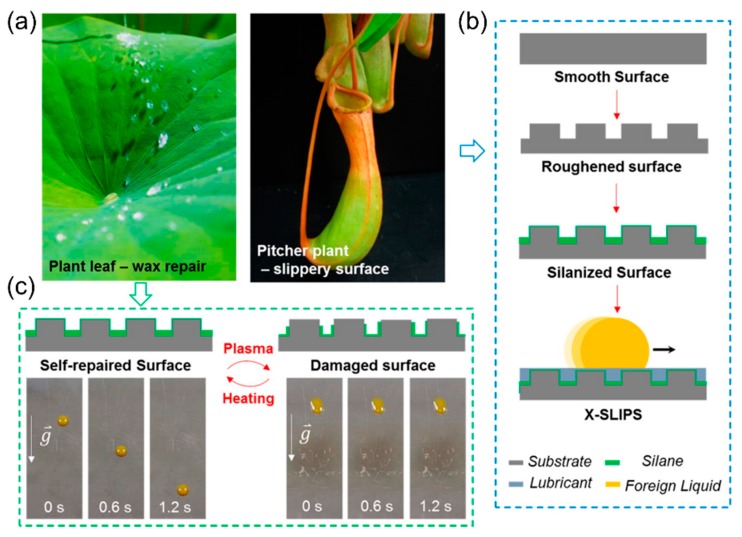
Bioinspired slippery liquid-infused porous surfaces (X-SLIPS) coating with thermal healing function. (**a**) Optical images showing a leaf of *Nelumbo nucifera* repelling water droplets (left) and a leaf of *Nepenthes* whose lamina is modified into a pitcher (right). (**b**) Schematic drawing that shows the *Nepenthes*-inspired fabrication process of a slippery coating. (**c**) Schematics showing the concept of self-repairing surfaces inspired by the wax repair of plant leaves compared with damaged surfaces (above). Liquid repellence of octane droplets on lubricated substrates is shown for undamaged and damaged silane coatings (bottom). Reprinted with permission from [63]. Copyright 2016 American Chemical Society.

Inspired by the wax repair of plant leaves and the slippery surfaces of the *Nepenthes* pitcher plants, Wang et al. [63] developed slippery liquid-infused porous surfaces (X-SLIPS) that can thermally heal even significant physical and chemical damage over large areas. These omniphobic coatings can be applied to numerous industrial materials of various shapes, such as metals, glass, plastics and ceramics. Figure 8 shows the plant models and the way that the biological findings have been abstracted to fabricate a cross-species bioinspired coating.

X-SLIPS coatings were created, first, by a micro/nanoscale texturing of the previously smooth surface. Subsequently, the textured surface was coated with perfluorinated silane resulting in a surface with highly hydrophobicity. Even a monolayer of silane is sufficient to serve as an intermediate layer between the textured solid and the liquid lubricant. After physical/chemical damage the silane molecule layer survives and the molecules serve as a source for the thermal self-repairing process. Finally, perfluorinated lubricant was applied to the chemically functionalized resulting surface, which then repelled a broad range of aqueous and organic fluids. The repair function was characterized by means of the restoration of the superhydrophilic nature of the surface after chemical damage; the self-repairing repeatability obtained by counting the number of successful rehealing cycles was dependent on the curing temperature applied and the robustness of the coating by adhesion and scratch tests [63].

Wang et al. [63] developed an X-SLIPS coating with self-repair function upon thermal stimulation, inspired by the wax regeneration and the slippery structures of various plant surfaces. Since the X-SLIPS are created by a combination of diverse functional principles from various plant models, they are not a simple blueprint. On the contrary, this is a clear indication for a systematic approach with a high degree of abstraction, as known from the biomimetic top-down approach or the problem-driven BID.

#### 4.1.4. Self-Sealing Foam Coating for Pneumatic Systems Inspired by *Aristolochia*

Tensairity^®^ is a lightweight construction for temporary buildings and consists of an air-filled membrane cylinder (air pressure between 100 and 500 mbar), a compression rod and two tension cables (wrapped around the cylinder by contra-rotating screwing), which make the so-called “air beams” flexurally rigid in their additional action. The technical question was whether a nature-inspired functional principle could be found for the rapid sealing of any cracks up to a diameter of 5 mm in the membrane of the pneumatic systems (Figure 6).

An appropriate biological role model was determined in the self-repair mechanisms in stems of the twining vines *Aristolochia macrophylla* and *Aristolochia ringens* [1,2,3,4,48,49,51]. Young axes of the lianas are self-supporting until they have found a suitable support structure such as a host tree or host branches. The necessary bending stiffness of these searching twigs is achieved by a closed ring of thick-walled and lignified sclerenchyma cells in the periphery of the stem cross-section. During secondary growth, tiny fissures occur in the ring of the strengthening tissues because of increasing radial and tangential stresses and strains caused by the increased secondary growth of tissues within the ring (xylem and phloem). As soon as a lesion arises, turgescent thin-walled and nonlignified parenchyma cells from the surrounding cortex tissue swell into the lesion and seal it. In this initial sealing phase, the cell walls of the sealing cells extend locally through viscoelastic/plastic deformation without observable cell wall synthesis [48]. The subsequent healing phase involves typical cell growth, cell division and partially, a lignification of the cell walls of the sealing cells. 

This rapid self-sealing process based on cellular plant structure has inspired the development of a biomimetic polyurethane coating consisting of closed foam cells [51]. Since no standardized test method existed for measuring and calculating the repair efficiency of pneumatic systems, a measurement method was developed as a side innovation that allowed the measurement of the air flow of membrane samples after they had been punctured with a nail of 2.5–5.0 mm in diameter. Based on the mass flow through the damage in a foam-coated membrane sample (*ṁ_coat_*) and the mass flow through a punctured but uncoated reference membrane (*ṁ_ref_*), the self-repair efficiency *R_flow_* can be calculated according to Equation (3):

(3) $$R_{flow}=1-\;\frac{{\dot{m}}_{coat}}{{\dot{m}}_{ref}}$$

The repair efficiency can be assumed to have values between 1 (complete repair) and 0 (no repair). With the help of this dimensionless value, we can compare the self-repair efficiency of coatings of various production methods, materials, layer thicknesses and types of injury [50]. For the test series, a polyvinyl chloride-coated polyester fabric (Ferrari Précontraint 1002, La Tour du Pin, France) was used as the membrane and subsequently coated with the molded foam RAKU-PUR 33-1024-3 (Rampf Giessharze GmbH & Co. KG, Grafenberg, Germany). Maximum repair efficiencies of 0.99 have been obtained for a low coating weight and thickness of the foam layer [50,52,53].

**Figure 9:**
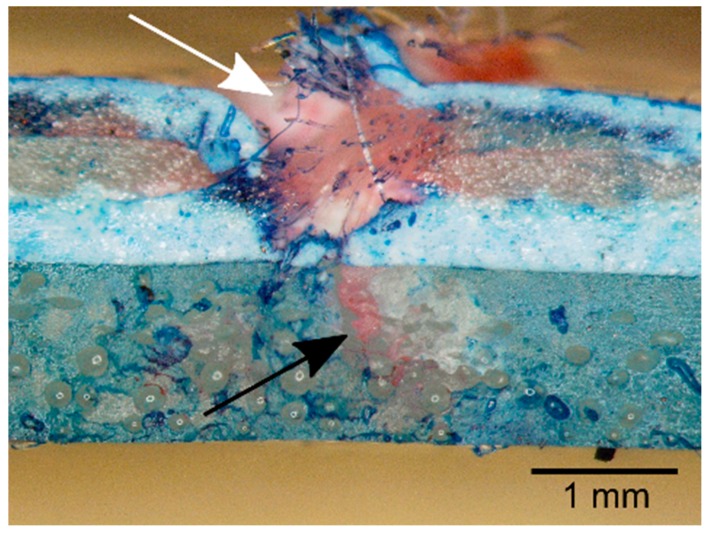
Cross-section of a test sample consisting of the fiber-reinforced membrane (top) and the foam coating (bottom). To improve the visibility of the structures, the surface was treated with a blue stamping ink. Before being used to puncture the membrane, the nail was dipped in red ink in order to make the puncture channel clearly visible. The membrane shows clear damage by the puncturing of the nail (white arrow), whereas the surfaces of the puncture channel in the foam (black arrow) lie on each other so precisely that the channel is recognizable only by the red color.

The simulation by using the finite element method and the results of the test series showed that the repair function in the coated membrane is a purely mechanical effect. The curvature of the inflated membrane samples favors the compression of the internal foam coating and results in a reduction of the damaged surface (Figure 9). At a given internal pressure and curvature of the membrane sample, the self-repair function is mainly determined by the thickness of the coating applied. Equation (4) describes the relationship between the strain *ε_x_* at the surface of the coating, which directly adjoins the membrane and the material and geometry parameters, where *p_i_* is the internal pressure, *r* the radius of curvature of the inflated membrane, *ν* the Poison’s ratio of the membrane material, *E_M_* the modulus of elasticity of the membrane material, *t_M_* the thickness of the membrane, and *t* the thickness of the foam coating [50].

(4) $$\varepsilon_{x}=\;\frac{1}{2}\;\frac{\rho_{i}\cdot r\cdot \left(1-v\right)}{E_{M}\cdot t_{M}}\;-\;\frac{t}{r}$$

The development of a self-sealing coating for Tensairity^®^ technology is a typical top-down approach of biomimetics (Figure 6). Without a doubt, the sealing cells of the *Aristolochia* species were the inspiration for the idea of choosing a closed-pore foam coating. However, unlike the biological models, in which individual sealing cells squeeze into the fissures that occur in the strengthening tissue, the sealing principle in the technical system is largely based on the reduction of the diameter of perforation in the foam layer favored by the compression of the foam coating on the inflated membrane, which always shows a curvature [49,50].

#### 4.1.5. Self-Healing Elastomers for Dampers Inspired by Latex-Bearing Plants

**Figure 10:**
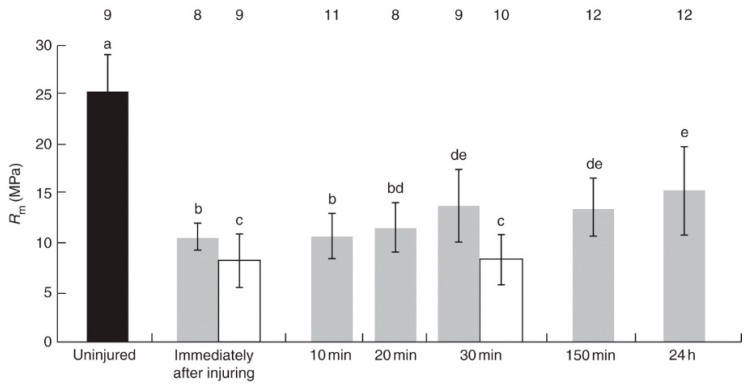
Self-sealing and self-healing in barks of *Ficus benjamina*. Tensile strength (*R_m_*) of uninjured bark (black columns) and at various times after damage (grey columns). White columns represent bark samples from which latex was cleaned immediately after injury. Numbers of samples tested are indicated above. Results marked with the same letter do not differ significantly. Reproduced from [73], by permission of Oxford University Press on behalf of the Annals of Botany Company.

Within the scope of self-repairing materials systems, latex-bearing plants such as the weeping fig (*Ficus benjamina*), the rubber tree (*Hevea brasiliensis*) and several spurge species (*Euphorbia* spp.) were selected as suitable models to meet the technical challenge of avoiding crack propagation. On the basis of mechanical tests, Bauer and Speck [73] analyzed the restoration of tensile strength in bark samples of *F. benjamina* as resulting from coagulation of latex during fast self-healing of fissures. Figure 10 shows that a significant increase in tensile strength occurs within 30 min after external injury, representing 55% of the value obtained for the uninjured bark. If the latex is removed, no self-healing can be detected within this time frame.

The coagulation mechanism of plant latex is well understood in *H. brasiliensis*. Microtubes (laticifers) contain emulsions with latex particles and membranous vesicles. An overpressure of up to 1.5 MPa is found within the laticifers [73,74]. After injury, a pressure drop occurs with the consequence that protein-containing membranous vesicles in the emulsion burst freeing their entrapped hevein proteins. These hevein proteins form (mediated by Ca^2+^ ions) dimmers that bind to protein-binding sites on the surface of the rubber particles so that, finally, coagulation of the latex particles takes place [83]. Analogous mechanisms are highly likely in *F. benjaminia*, as was established by various physical and chemical examinations [75]. 

**Figure 11:**
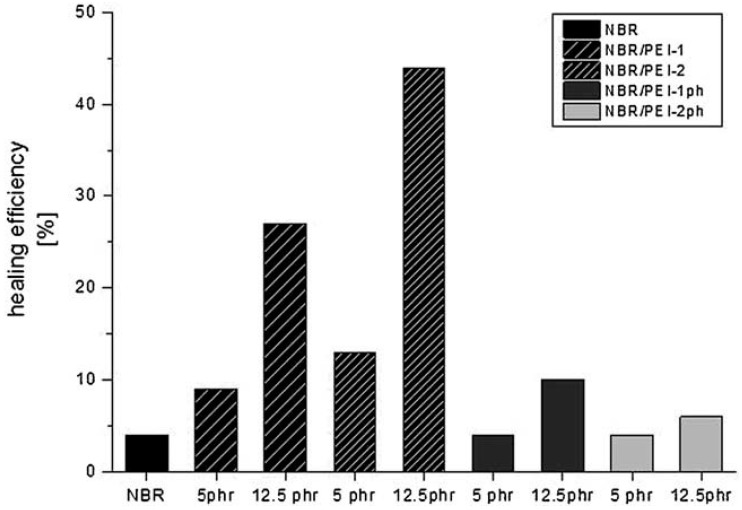
Self-healing efficiency of pure nitrile butadiene rubber (NBR) strips and strips made of various types of microphase separated NBR/polyethyleneimine (PEI) blends after the strips were cut in half, re-joined under compression and subsequently annealed for 12 h at 100 °C and stored for 12 h at room temperature. Best self-healing efficiency was obtained with NBR/PEI-2 blends with a high PEI content (12.5 phr) of unmodified PEI with high molecular mass (2000 g mol^−1^). Reprinted from [76] by permission of John Wiley & Sons, Inc.

Based on this self-healing mechanism, two approaches were used for producing novel bioinspired self-healing elastomers showing significant mechanical restoration after a macroscopic cut. First, inspired by the function of Ca^2+^ ions, ionomeric self-healing elastomers were designed in which self-repair works well in the nonvulcanized state but not if vulcanization is completed [75]. In a second approach, microphase separated nitrile butadiene rubber (NBR)/hyperbranched polyethyleneimine (PEI) blends were developed that chemically mimicked microcapsules with self-healing agents without a hull by phase separation. A maximum self-healing efficiency calculated for a restored tensile strength of 44% could be achieved for various PEI molecular architectures (Figure 11) [76,77]. 

To sum up, the development of the self-healing elastomers is a typical top-down approach that was driven by the search for bioinspired elastomer chemistries/blends that efficiently heal (micro)cracks and, by these means, that keep fissure under a length critical for system failure. From the approaches tested, microphase separated NBR/PEI blends show by far the highest potential, even in the vulcanized state.

### 4.2. Animals as Idea Providers or Concept Generators

#### 4.2.1. Self-Healing Composites Inspired by Nacre

In recent years, nacre has attracted enormous attention because its brick-and-mortar architecture of stiff inorganic constituents (95 vol. % interlocking aragonite platelets) and a soft organic matrix (5 vol. % protein–polysaccharide) leads to an extraordinary combination of mechanical properties such as stiffness, toughness and strength. Based on crack morphologies, Feng et al. [84] found that crack deflection, fiber pull-out and organic matrix bridging are the three main toughening mechanisms acting on nacre. In contrast to man-made hard materials, natural nacre is designed for absolute fracture resistance [21]. Produced by some molluscs, nacre has inspired various research groups to develop synthetic nacre-like materials with similar fascinating properties, such as the combination of stiffness and toughness leading to robust structural ceramics.

Zhu et al. [64] generated nacre-mimetic films composed of dynamic polymers that had a low glass-transition temperature, that were bonded by quadruple hydrogen-bonding motifs and that were subsequently assembled with high-aspect-ratio synthetic nanoclays. Because of their high dynamics, all so-called EG-UPy-()-polymers immediately and autonomously self-heal after a cut or rupture and when implemented into nacre-inspired nanocomposites, they at least allow the healing of nanovoids and nanoscopic cracks.

**Figure 12:**
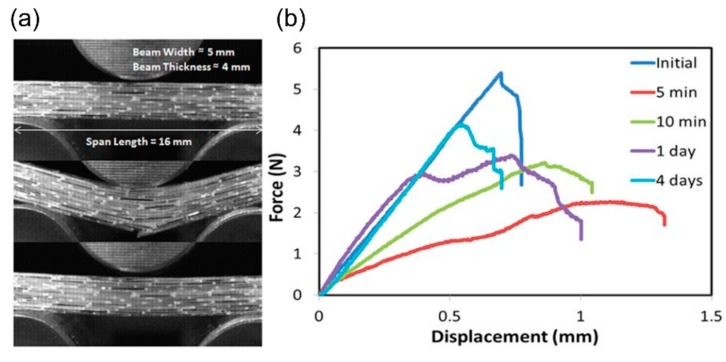
Three-point bending test of the glass–polymer composites with thin glass bricks. (**a**) Images of test samples before (top), during (middle) and after (bottom) fracture. The bottom picture shows that the composite autonomously had reached the initial configuration (top). (**b**) Force–displacement curves of composites. After the test to fracture the samples were left healing at room temperature for various amount of times. Reproduced from [62], CC BY 4.0 [85].

D’Elia et al. [62] developed an autonomous nacre-like material with self-healing function. The authors describe natural nacre as being composed of stiff blocks bound together using thin interfacial soft layers that can also provide sacrificial bonds for self-repair. Inspired by this healing mechanism they created a simple design idea able to heal repeatedly without degradation or external stimuli. The nacre-inspired materials system consists of hard, inorganic glass bricks and thin layers of the supramolecular polymer poly(borosiloxane) well-known for its self-healing capabilities (Figure 12).

In 2017, Hwang et al. [61] presented another nacre-inspired material whose self-healing function, in contrast to the previous examples, is not autonomic but must be triggered by heat. The newly designed strong, tough and self-healing composite is synthesized by universal spherical building blocks. The composite system consists of calcium silicate porous nanoparticles (CPNPs) with unprecedented monodispersity over particle size, particle shape and pore size enabling their effective loading and unloading with organic sealants. The heating of the damaged composites also triggers the controlled release of the nanoconfined sealant into the damage site, thereby allowing a moderate recovery of strength and toughness.

According to Wegst et al. [21] natural nacre is designed for absolute fracture resistance or in other words this highly mineralized composite material arrests crack propagation and avoids catastrophic failure. Thus, a precise closing of a macroscopic crack in natural nacre is hardly possible because, in this case, all platelets would have to be pushed back to their original location or brick fracture has to be fully repaired. The situation is different for nanosized cracks in the organic interphase because the healing of subcritical defects of the organic phase of natural nacre can be reasonably expected. Therefore, the repair of nanovoids is more feasible. In addition, nacre-inspired materials systems with self-healing effects could be developed if further functional principles also from other role models are combined according to the concept of *going beyond biology* [2]. Thus, nacre-inspired materials systems that take into account the self-assembly of organic–inorganic composites and nano/microscale mechanisms may lead to highly damage-tolerant bioinspired materials. The three presented examples of nacre-inspired materials systems have to be considered on this background. Although no or only vague information about the selected principles and the flow of ideas from the biological role model to the self-healing technology is presented in the relevant publications themselves, the developments can be considered as bioinspired, even though they mainly refer to the increase in toughness through an architecture mimicking that of nacre.

#### 4.2.2. Self-Healing Materials Inspired by Mussel Byssus 

**Figure 13:**
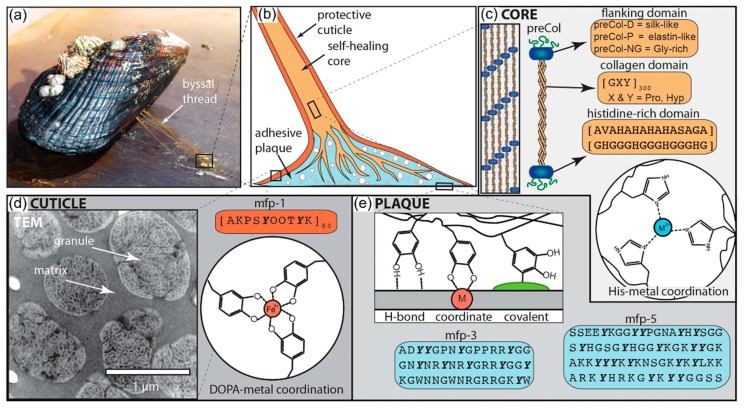
Structural hierarchy of the mussel byssus. (**a**) Mussels anchor onto surfaces by means of a byssus. (**b**) Individual byssal threads consist of a fibrous core, which is protected by a thin cuticle and ends in an underwater adhesive plaque. (**c**) The core is comprised of collagenous modular proteins, referred to as prepepsinized collagens (preCols), which are organized into a semicrystalline framework and stabilized by histidine–metal coordination bonds. (**d**) The cuticle, which resembles a particle-reinforced composite, comprises mainly a repetitive 3,4-dihydroxyphenylalanine (DOPA)-rich protein, mfp-1, which is cross-linked via metal coordination bonds with Fe^3+^. (**e**) Plaque adhesion is mainly mediated by DOPA and Lys-rich proteins mfp-3 and mfp-5, which utilize DOPA to create a range of interactions with surfaces. In the protein sequences above, bold and italicized Y represents DOPA. Reprinted from [67] by permission of John Wiley & Sons, Inc.

A variety of studies has been carried out on biological fibers such as the byssal threads of marine mussels (*Myrtilus* sp.), belonging to the phylum Mollusca. A typical byssus consists of 50–100 individual byssal threads, each 2–5 cm long. Mussel byssal threads are completely acellular and almost entirely composed of proteins consisting of three parts: a shock-absorbing fibrous core, sheathed by a thin protective cuticle and ending in an underwater adhesive plaque (Figure 13). The threads securely anchor the mussels to wave-battered surfaces on rocky seashores [67]. Mussel byssal threads were tested in subsequent loading cycles. The molecular model of reversible deformation in the core indicates that, if under a critical load is exceeded, sacrificial bonds in a portion of the extensible domains break successively and a hidden length is unfolded, resulting in a total elongation of up to 50% of the nanofibrils. After unloading, a full recovery of the structure is observed in the sense of a refolding but not with regard to its mechanical properties. After a long-term recovery, however, healing is also completed at a molecular level by cross-linking the sacrificial bonds and thus achieving full recovery of the mechanical properties [3,68]. Schmitt et al. [69] have shown that mechanically active protein–Zn^2+^ cross-links in the distal thread core play a crucial role during thread self-healing.

The successful translation of mussel-inspired functional principles into various synthetic materials with self-healing effect have been made by several research groups. Ahn et al. [66] have developed a mussel-inspired polymer that heals damaged sites under wet or moist conditions. Wet self-mending of synthetic polyacrylate and polymethacrylate materials that are surface-functionalized with mussel-inspired catechols is initiated and accelerated by hydrogen bonding between interfacial catechol moieties and consolidated by the recruitment of other noncovalent interactions contributed by subsurface moieties. Since the healed and the pristine specimens show similar mechanical properties such as tensile strength, we can assume that the healing under water is triggered by the formation of extensive catechol-mediated interfacial hydrogen bonds. In 2013, Kogsgaard et al. [70] presented the development of a self-healing multi-pH-responsive hydrogel inspired by mussel adhesive proteins. DOPA was attached to an amine-functionalized polymer. This creates a multireactive system when reacting with iron. Since the degree of cross-linking is pH controlled, a bistable gel system is obtained. The shift in pH from acidic towards basic values leads to a self-healing high-strength hydrogel.

In summary, the mussel-inspired synthetic materials closely resemble the self-healing principles found in the biological acellular protein structures. Obviously, in addition to the biomimetic applications described above, biotechnological efforts to produce new materials from protein and peptide building blocks have also been developed [67] but these are not presented in this overview.

#### 4.2.3. Self-Healing Concrete Mimicking the Bone Healing Process

Inspired by the self-healing of spongious bones, Sangadji and Schlangen [71] presented a concept for the self-healing of concrete in 2013. They clearly showed the comparability of wound healing in bone with the three phases: (i) inflammatory response and blood clotting; (ii) cell proliferation and matrix deposition; and (iii) matrix remodeling leading to damage healing in synthetic systems with three analogue phases, namely (i) actuation (triggering); (ii) transport of self-healing agents; and (iii) chemical repair. Moreover, bone morphology was imitated by using a prefabricated cylindrical porous concrete core that was placed inside a concrete beam. The specimens with a notch in the middle of the sample height were tested under tensile loading. Occurring cracks were healed with an expoxy-based agent injected manually by using a syringe. In addition, a fluorescent dye was used to highlight crack sealing. To guarantee epoxy polymerization, the samples were kept in the oven at approximately 35 °C for 24 h. 

**Figure 14:**
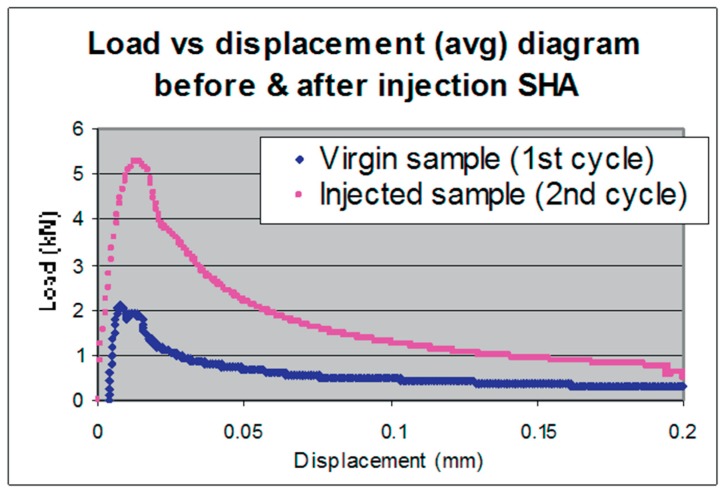
Average tensile load–displacement diagram of a virgin (blue curve) and a healed concrete sample after injection of a self-healing agent (SHA) (red curve). Reprinted from [71], Copyright 2013, with permission from Elsevier.

Figure 14 shows the average load–displacement curve of a virgin sample with a peak tensile load value of around 2.2 kN, during a crack mouth opening displacement (CMOD) reaching 15 µm. This is followed by a nonlinear softening behavior up to a CMOD of 200 µm. The second tensile test of the healed sample led to a similar curve but the peak value reached 5.2 kN at a CMOD of 25 µm. This higher mechanical value can be explained as follows: the injection of the self-healing agent results in a (mechanically superior) polymer–cement composite with all pores filled.

Since the authors dedicate an entire chapter explicitly to the developmental concept and the comparison between self-healing mechanisms in nature and synthetic systems, we can assume that the development of the self-repairing concrete is a top-down approach in biomimetics.

#### 4.2.4. Self-Healing Composites Inspired by Hemostasis

Composite materials are increasingly used for load-bearing components in aerospace constructions. Damage in terms of delamination, debonding or matrix cracking, which can often be difficult to detect, ultimately lead to catastrophic failure scenarios that can be mitigated by self-healing solutions, among other measures [13,56,79,80]. Based on these technical challenges, self-healing mechanisms have been developed for the composite materials presented below. 

**Figure 15:**
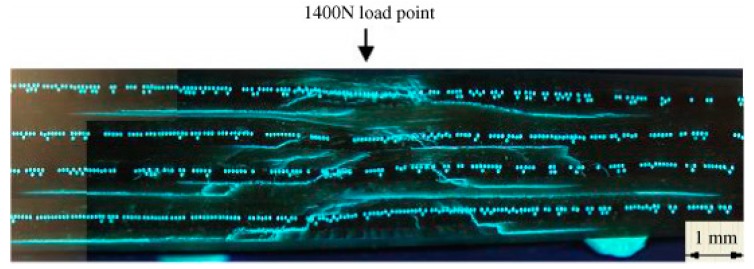
Released ultraviolet (UV)-fluorescent “healing agent” infiltrates the damage zone after flexural strength testing. Reproduced with permission from [81]. Copyright 2006, IOP Publishing Ltd.

In 2005, Pang and Bond [56,80] presented the so-called “bleeding composites” that have a self-repairing function and that were developed in analogy to the biological healing process in living organisms. The functional principle of the presented bleeding action from liquid-filled hollow fibers includes, first, the release of a florescent dye that contributes to the visual enhancement of the damage site, and, second, the discharge of a healing agent that infiltrates the damaged area and that can significantly contribute to the restoration of the mechanical properties. In this particular case, the fluorescent dye and the healing agents (resin and hardener) are stored in hollow glass tubes added as plies to the composite. After four-point bend flexural testing, these composite systems show a strength restoration of up to 97% [56] and 93% [80] compared with the pristine state, depending on the healing assessment and healing treatment. Here, healing efficiency has been calculated according to Equation (1). In 2006, Trask and Bond [81] developed a system of hybrid solid/hollow glass fiber-reinforced epoxy in which the healing fibers were only positioned at the most critical interfaces (Figure 15). 

In the subsequent years, composite systems with self-healing effects were further developed (i) with regard to various healing agents packaged in microcapsules, hollow fibers or vascular networks [13], and (ii) in the direction of industrially relevant applications [79]. Luterbacher et al. [79] presented a feasibility study on stringer run-out panels with embedded vascular microchannels in 2016. The decisive advantage, however, is that even larger areas of damage can be addressed with such vascular systems, as the healing agent can be supplied from a larger external reservoir. Compared with the baseline specimens, the introduction of microchannels reduces strength by 15%, whereas stiffness is reduced by only 4%. The specimens were tested under tensile loading. The healing efficiency of delamination was calculated by using Equation (1) comparing the stress level at which the delamination initiated at the tip of the healed and pristine strap, respectively. Dependent on the healing agent, the stress level of the healed specimens increased, which led to a healing efficiency ranging between 137% and 145%. This can be explained by an increase in the resin layer thickness. The healing efficiency of stiffness was calculated by using Equation (2) based on the comparison of the respective elastic moduli. The stiffness was completely restored with healing efficiencies of 98% and 99%.

Certainly the “bleeding composites” have been inspired and developed by analogy with the self-healing of living beings. In 2011, Norris et al. [82] summarized the flow of ideas from nature’s solutions to engineering solutions for fiber-reinforced composite systems in four stages. First, the delivery of the healing agent: in nature healing agents are delivered by vascular tissues. In technology, healing agents are dispersed in the matrix or stored in microcapsules, hollow fibers or vascular networks. Second, reaction to damage: natural reactions include hemostasis and compartmentalization in terms of boundary layers. In synthetic composites, the fracture of vessels leads to a release of the healing agent, which is transported from an external reservoir to the damage site. Third, healing processes: nature’s inspiration comes from inflammation, proliferation and remodeling after skin wounds and the continuous remodeling of bones. Fourth, damage visualization by, for example scabbing, bruising and scarring in nature and by dyes in synthetic materials.

### 4.3. Living Organisms as Idea Providers or Concept Generators

#### 4.3.1. Self-Healing Polymer Systems Inspired by Secretion

**Figure 16:**
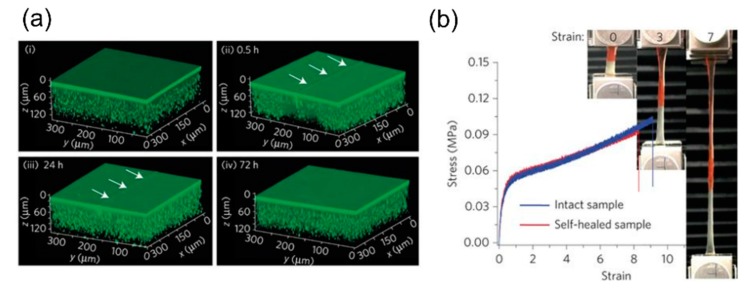
Self-healing of a droplet-embedded polymer–gel matrix. (**a**) Three-dimensional confocal fluorescence images before damage (i) and after damage at 0.5 h (ii), 24 h (iii) and 72 h (iv). Arrows highlight the damaged region. (**b**) Stress–strain curves of the intact and self-healed urea and polydimethylsiloxane (uPDMS)_2_-based sample. Inset images show the self-healed sample composed of two pieces under different strains (one piece was dyed red for contrast). (**a**,**b**) The liquid surface layer had a thickness of 200 ± 20 µm. The silicone oil loading was 160 wt %. Reprinted by permission from Springer Nature Customer Service Centre GmbH: Springer Nature [65], Copyright 2015.

Secretion, an omnipresent phenomenon in living organisms, describes the movement of a material from one place to another. Inspired by living secretion systems, Cui et al. [65] have presented a self-regulating secretion system that consists of liquid-storage compartments in a supramolecular polymer–gel matrix with a thin liquid surface layer. Since local material damage induces the secretion of the stored liquid through feedback between polymer cross-linking, shrinkage of the shell-less droplets and liquid transport, this material has a self-healing function. Cui et al. [65] have designed copolymers of urea and polydimethylsiloxane (uPDMS), whose urea units are reversibly cross-linked by hydrogen bonding. A droplet-embedded material is formed from a homogeneous solution of uPDMS, silicone oil and tetrahydrofuran by the phase separation of oil droplets in the micrometer range. Finally, the oil droplets are immobilized by gelation in the bulk. With successive secretion cycles, the liquid droplets shrink continuously and the initially opaque gel with droplets becomes increasingly transparent. Full transparency is correlated with the complete consumption of the droplets, the loss of secretion capacity and consequently, the ability to self-heal. Figure 16 shows visualizations and the mechanical performance of intact and self-healed droplet-embedded material.

During evolution, bacteria and eukaryotic cells have developed completely different secretion mechanisms and a multitude of secretory functions. Unfortunately, Cui et al. [65] do not specify which model was used for the development of their self-healing droplet-embedded materials. We assume that a technical question was posed at the beginning of the development of the self-healing polymer. Obviously, natural secretion was the model. Therefore, the product can be regarded as bioinspired.

## 5. Discussion

Interest in the development of technical self-repairing materials was aroused by the paper of White et al. [55] that appeared in *Nature* in 2001. It triggered a wave of scientific projects, applications and conferences that continues until today. The forward-looking approach of White and his co-authors has spurred on several bioinspired and biomimetic solutions. However, astonishingly, almost 20 years later, only a few bioinspired materials with self-repair function have been developed, with even fewer having reached the market place.

Although all scientists acknowledge that self-repair has been and remains a success factor for biological evolution, only a few scientists and engineers are systematically working on the transfer of knowledge from biological to technical materials systems as part of interdisciplinary research projects. On the one hand, many details of self-repair in plants and animals are known but have not yet been transferred into technical applications. On the other hand, because of obvious analogies between natural and technical solutions, technical applications are retrospectively interpreted as bioinspired or biomimetic. The latter probably not least because of the normative and emotional aspects of bioinspiration and biomimetics [10]. Nevertheless, these analogies might be a starting point for further basic and applied research on biology-derived improvements or adaptations of previously developed materials or even new developments of self-repairing materials. Analogies and reinterpretations in the sense of an *a posteriori biomimetization* are the consequence of the lack of a design language that unambiguously describes the possible origin from a biological role model and that gives additional information about the type of knowledge transfer. Therefore, for a clear classification it is necessary to collect background information about the development of products by means of literature researches and/or interviews of the participating scientists [10].

Learning from nature is often linked with the hope of learning from solutions that seem to have been optimized during biological evolution. Precisely because of the flow of ideas and of functional principles from nature into technology, bioinspired and biomimetic solutions are considered to have great potential to contribute to more sustainable development. In this context, von Gleich has coined the term “biomimetic promise”, which implies that biomimetic products and technologies possess an exceptional quality [86,87]. In addition to the normative aspect of a contribution to sustainability, emotional aspects such as feelings, moods and fascination also come into play [10]. Especially with self-repairing materials, however, the picture is ambivalent. On the one hand, we desire products that have a long service life and thus contribute to conserving resources and to avoiding waste. On the other hand, indestructible products accumulate and can become an environmental problem. Nevertheless, scientists, engineers and customers transfer their fascination of natural systems and their reverence for life to bioinspired and biomimetic products in general and to bioinspired and biomimetic self-repairing materials in particular [10]. A closer look reveals that bioinspired and biomimetic products do not automatically contribute to sustainability. Therefore, each individual case must be investigated, whereby the selection of the comparison system is of crucial importance [4,88,89].

A further question to be critically discussed is whether one can even speak of a bioinspired or biomimetic material, if the principle of self-repair is based solely on the presence of a chemical reaction (e.g., hydrogen bonds). On the one hand, this seems more an expression of common chemical and physical laws that apply in all natural sciences and engineering. On the other hand, this fact is precisely the legitimation for biomimetics, which would not be applicable without such a common basis. Furthermore, we have shown that biomimetics is not a question of scaling, as it can take place at all hierarchical levels.

In summary, the subdivision into a rapid sealing and a longer-term healing phase can be said to apply to both natural and synthetic systems. Table 6 shows a compilation of self-repair principles that have been applied to synthetic materials to date.

Finally, both aspects of the concept of damage control, namely damage prevention and damage management, will be discussed in relation to biological, bioinspired and biomimetic self-healing systems. Damage control is associated with the mechanical properties stiffness and strength. If a material is loaded exclusively in the elastic range, it can return to its preloading configuration without showing any plastic deformation [5]. The greater the range of elastic loading, the greater the probability that the material will not be overcritically loaded. Designs for damage prevention also include material with high strength. Strong materials show higher critical load or loading time before disintegration or crack formation. In addition to these material properties, damage to structures can be avoided by, for example, adaptive reactions to mechanical stresses such as reaction wood or thigmomorphogenesis and resistance reduction by reconfiguration or gradual transitions to avoid stress peaks and thus predetermined breaking points.

However, if damage occurs to a material, it remains forever or has to be repaired. At the material level, repair means that mobile agents arrive to fill the gap caused by the damage and connect the fracture surfaces [5]. Designs for damage management include not only self-sealing and self-healing of the damage site but also the development of a predetermined breaking point for discarding of senescent plant parts, such as leaves, fruits and seeds (abscission) or the reflex separation of animal appendages, such as limbs, tails or arms (autotomy).

In living nature, both damage prevention and damage management occur as a matter of course. This double protection (redundancy) against damage guarantees the maintenance of the functional capability of the entire system (resilience and resistance) and finally its survival. Damage prevention and damage management should therefore not be regarded as separately acting either/or aspects but as complementary design concepts of materials and structures. This is also likely to lead to greater acceptance by future users of the new synthetic materials systems.

## 6. Conclusions

Sealing and healing of wounds can be seen as a fundamental function of living nature evolved independently and several times in plants, animals and all other groups of organisms during the 3.8 billion years of biological evolution. These self-repair mechanisms are a treasure trove in which biologists and engineers have become increasingly interested. The biologically inspired design and the biomimetic approach have emerged as the best-known and most used methods to systematically transfer biological insights into technical applications. In recent years, however, only a few self-repairing materials systems based on biological models have been developed that can seal or (partially) heal damage and restore the respective mechanical properties and structural integrity compared to the intact sample. Interestingly, only a few examples are marketable products but often the solutions presented are design ideas developed on a laboratory scale.

## Figures and Tables

**Table 1:** Characteristics of self-repairing, self-sealing and self-healing valid for biological and technical materials (adapted from [3]).

Self-Repairing =	Self-Sealing	+	Self-Healing
Fissures …	…are sealed rapidly.		…are healed over a longer time span.
	…are still present.		…are no longer present.
	…are repaired functionally.		…are repaired structurally.
	…are not repaired in terms of mechanical properties.		…are repaired (at least partially) in terms of mechanical properties.

**Table 2:** Overview of common self-repair mechanisms found in vascular plants arranged according to increasingly smaller hierarchy levels.

Hierarchy Level	Self-Sealing	Self-Healing
Organs	Bending or contraction of organs	Regeneration in terms of recreating entire organs
Tissues	Rolling in, overlapping or hook-like deformation of epidermal tissue at the wound edges	Formation of a wound periderm including cell division
Cells	Parenchyma cells swell into fissures	Lignification of cell walls Formation of a (ligno-)suberized boundary layer in the cell walls
Molecules	Discharge of plant sap (mucilage, latex, resin)	Coagulation of latex

**Table 3:** Overview of common self-repair mechanisms found in human wound reaction arranged according to increasingly smaller hierarchy levels.

Hierarchy Level	Self-Sealing	Self-Healing
Organ systems	-	-
Organs	-	Regeneration in terms of recreating entire organs (e.g., angiogenesis)
Tissues	Vasoconstriction	Filling the gap by formation of tissue (e.g., granulation tissue) Epithelialization
Cells	Blood clotting Clearance of wound from pathogens	Contraction of the wound edges (e.g., myofibroblasts) Apoptosis
Molecules	Deposition of temporary extracellular matrix (e.g., fibrin, fibronectin)	Formation of extracellular matrix (e.g., cellulose, elastin)

**Table 4:** Biomimetic approaches: individual steps [42,43].

Steps	Top-Down Approach = Technology Pull Process	Bottom-Up Approach = Biology Push Process
Step 1: Scientific question	An application-oriented question is asked in order to solve a particular technical challenge.	A basic research-oriented question is asked in order to gain knowledge about biological systems.
Step 2: Biological concept generator	Within the scope of a screening process, initially relevant criteria are defined and then suitable biological role models are selected that are quantitatively analyzed in terms of their morphological and anatomical structure and mechanical properties.	
Step 3: Functional principle	The results of the in-depth investigations of the biological model lead to the underlying functional principle being deciphered.	
Step 4: Abstraction	The functional principle is translated into a common language understood by natural scientist and engineers, such as functional models, construction plans, circuit diagrams, and numerical and analytical models.	
Step 5: Technical application	Based on the abstracted description, feasibility studies are carried out and samples at the laboratory scale, prototypes and pilot series can be produced.	
Step 6: Biomimetic product	Here, the transition from laboratory and pilot scale to commercial production is made.	

**Table 5:** Chronological overview of key publications concerning nature-inspired self-repairing materials.

First Published in Year	Inspired by	Self-Repair in Biological Role Models	Knowledge Transfer from Living Nature to Technology	Bioinspired or Biomimetic Material with Self-Repair Function	Bioinspiration, BID or Biomimetic Approach ^1^	Key References/Section
2018	Plants	Leaves of *D. cooperi*	Release of stored elastic energy	Phase-separated polymers with built-in shape-memory effect leading to self-healing	Bottom-up approach	[44,45,46,47]; Section 4.1.1.
2018	Humans	Epidermis of human skin	Hierarchically stratified structure of a soft inner and a hard outer material layer	Hierarchical coating system of hybrid multilayers with synergetic self-healing function	Top-down approach or problem-driven BID	[58]
2018	Plants	Follicles of the plant genus *Banksia*	Waxes at the suture of the two valves protecting the seed seal up microfissures	Wood platelets sealed by carnauba wax	Bottom-up approach or solution-based BID	[59,60]; Section 4.1.2.
2017	Animals	Architecture of nacre	Hierarchical structure	Heat-triggered composites releasing sealant	Bioinspiration	[61]; Section 4.2.1.
2016	Animals	Architecture of nacre	Sacrificial bonds in organic layer	Autonomous self-healing layers of supramolecular polymer	Bioinspiration	[62]; Section 4.2.1.
2016	Plants	Surfaces of plant leaves (*Lotus*) and *Nepenthes* pitcher plants	Wax repair of leaves and slippery surfaces of pitfall traps	Self-repairing slippery liquid-infused porous surfaces (X-SLIPS)	Top-down approach or problem-driven BID	[63]; Section 4.1.3.
2015	Animals	Architecture of nacre	Self-assembly	Self-healing polymers with high dynamics	Bioinspiration	[64]; Section 4.2.1.
2015	Living nature in general	Living tissues with self-regulated release systems of liquids	Continuous, dynamic, liquid exchange between shell-less droplets, matrix and surface	Self-healing droplet-embedded gel material	Bioinspiration	[65]; Section 4.3.1.
2014	Animals	Byssal threads of marine mussels (*Myrtilus* sp.)	Self-assembly	Wet self-mending polymers, surface-functionalized with catechols	Top-down approach or problem-driven BID	[66]; Section 4.2.2.
2013	Animals	Byssal threads of marine mussels (*Myrtilus* sp.)	Metal coordination-based cross-linking of proteins	Self-healing, multi-pH-responsive hydrogel	Top-down approach or problem-driven BID	[67,68,69,70]; Section 4.2.2.
2013	Humans	Hemostasis of spongious bone	Delivery and reaction of healing agents in a porous concrete core	Self-sealing and self-healing concrete	Top-down approach	[71]; Section 4.2.3.
2011	Plants	Stems of twining liana (*Aristolochia* sp.)	Sealing cells squeezing into tissue fissures	Self-sealing closed cell polyurethane foam coating for pneumatic systems	Top-down approach	[48,49,50,51,52,53]; Section 4.1.4.
2011	Plants	*Nepenthes* pitcher plants	Liquid-repellent microtextured surfaces with a stable air–liquid interface	Self-healing, slippery, liquid-infused porous surface(s) (SLIPS)	Top-down approach or problem-driven BID	[72]
2010	Plants	Latex-bearing plants	Latex discharge and latex coagulation	Self-healing elastomers for dampers	Top-down approach	[73,74,75,76,77]; Section 4.1.5.
2010	Plants	Waxy surfaces of plant leaves (*Lotus*)	Superhydrophobicity by regenerating the epicuticular wax layer	Self-healing superhydrophobic coatings	Top-down approach or problem-driven BID	[78]
2005	Humans	Hemostasis and bone healing	Delivery and reaction of healing agents	“Bleeding composites” for aerospace applications	Top-down approach or problem-driven BID	[13,56,79,80,81,82]; Section 4.2.4.

^1^ Explanations are given in Section 3.

**Table 6:** Overview of bioinspired self-repair principles found in technical self-sealing and self-healing systems.

Self-Repair Principles		Examples	References
Self-sealing	Agent release	Wax	[59,60]
		Resin and hardener	[13,56,79,80,82]
	Deposition of material	Filling pores	[61,71]
		Secretion	[65]
	Deformation	Shape-memory	[45]
	Mechanical stress	Compression	[50,52,53]
Self-healing	Chemical reactions	Polymerization	[71,75,76,77]
		Metal–ligand coordination	[66,70]
		Hydrogen bonding	[64]
		Dynamic boron–oxygen bonding	[62]
